# The Proper Mode of Vaccinating

**Published:** 1881-03

**Authors:** Barton Dozier

**Affiliations:** Ukiah City, Cal.


ARTICLE II.

### THE PROPER MODE OF VACCINATING.

BY BARTON DOZIER, M. D., UKIAH CITY, CAL.

As there seems to be considerable diversity of opinion among physicians as to the "modus operandi" in vaccinating in order to obtaining the best results, I will laconically describe the manner in which I have performed this operation during a recent threatened small-pox epidemic in this community ; and with what success.

My percentage of successful vaccinations so far surpassed my expectations, and also that of my colleagues, who used the same virus as myself, but a different mode of scarifying the surface, that I have concluded failures, in vaccination, should be more frequently attributed to an improper mode of scarification, than to poor or ineffective virus, or a " peculiar condition of the system."

I use *only fresh bovine virus ;* and prefer the ivory points to any other. I dip the point to be used in *cold water*, then lay it aside, in order that the virus coating may become well dissolved or softened. In the interim I scarify with a dry ivory point, stroking very lightly, scraping off as completely as possible, *without bringing blood*, the cuticle or scarf skin, for a space of about a quarter of an inch square. I then rub over the abrasion the previously prepared point, until the virus is all off, and insist on the sleeve being kept up for at least five minutes after the operation. I then place over the part a piece of adhesive plaster of sufficient size to cover well the scarification. Out of two hundred and twenty-five vaccinated adults and children, and many of the former having been successfully vaccinated before, I got a result of *ninety-seven* per cent. of successful vaccinations ! While my colleagues, who used lancets, principally, with which to scarify and *cut* rather than *scrape* through the outer skin, consequently causing more or less bleeding, were successful in not more than fifty per cent. of their cases.

